# Gendered Attributions of Blame and Failure to Protect in Child Welfare Responses to Sexual Abuse: A Feminist Critical Discourse Analysis

**DOI:** 10.1177/10778012211024263

**Published:** 2021-09-13

**Authors:** Corry Azzopardi

**Affiliations:** 1 Suspected Child Abuse and Neglect Program, Division of Paediatric Medicine, 7979 The Hospital for Sick Children, Toronto, ON, Canada; 2 Factor-Inwentash Faculty of Social Work, 7938 University of Toronto, Toronto, ON, Canada

**Keywords:** child sexual abuse, child welfare, failure to protect, nonoffending mothers, critical discourse analysis

## Abstract

Gender-based relations of power and attributions of blame for child sexual abuse have been longstanding in child welfare policy and practice. Nonoffending mothers continue to be ascribed responsibility through the ideologically and institutionally entrenched doctrine of failure to protect. Feminist critical discourse analysis was used to (a) expose and disrupt dominant discourses of gender, motherhood, and risk that operate to construct and reinforce notions of blame and failure to protect, as enacted by way of child welfare text in context; and (b) build a credible case for social and organizational change grounded in an alternative discourse with greater explanatory power. Progressive avenues for resistance, negotiation, and transformation are proposed.

## Introduction

Blame for child sexual abuse (CSA) has historically been attributed to nonoffending mothers, at least in part, by reason of complicity or negligence (Azzopardi et al., 2018). Although overt insinuations of conscious or unconscious maternal collusion have diminished since early psychoanalytic and family systems theory eras, the legacy of mother-blame lives on in gendered child welfare policies and practices through the contemporary doctrine of failure to protect (FTP). A widely adopted “commonsense” principle and statute, FTP is grounded in the assertion that caregivers have a moral and lawful duty to protect the child in their care from avoidable harm, deeming those who fail to fulfill this duty as liable for the resulting harm or risk of harm. Most commonly enacted in cases of sexual and domestic violence, FTP standards are firmly entrenched in child welfare and criminal justice systems, have a disproportionate effect on women, and come with serious social and legal repercussions (Strega et al., 2013).

Child protection service (CPS) substantiation of FTP occurs in the minority of CSA investigations and generally involves a determination of abuse by omission or supervisory neglect, whereby a caregiver knew or should have known of the risk of abuse, yet failed to take reasonable action to protect the child (Coohey, 2006). The criteria applied in making such decisions have weak scientific grounding, lack clear operationalization and uniform application, and impact racialized women disparately (Henry et al., 2020; Shadoin & Carnes, 2006). Judgments of FTP are immersed in the abstract and contested concept of risk aversion, a fundamental organizing principle in child welfare (Swift & Callahan, 2009). State mandates to protect children from harm and risk of harm demand appraisal of caregiver capacity to protect. When there are insufficient legal grounds to remove an offender from a victim's environment, as holds true much of the time, the burden of protection is placed upon CPS and often delegated to the primary nonoffending caregiver. Women, mostly biological mothers, comprise 90% of those identified as primary caregivers in child maltreatment investigations, irrespective of sole-parent or multiparent household demographics (Fallon et al., 2020). With little purposeful engagement with fathers, caregiver capacity to protect tends to be synonymous with maternal capacity to protect in a system where gender-biased policies and practices have been pervasive and enduring (Scourfield, 2006; Scourfield & Coffey, 2002).

Mother-blame for CSA has been longstanding in child welfare (Breckenridge & Baldry, 1997). It has been shown that mothers are classified as offenders or co-offenders in nearly half of CSA cases investigated by CPS, compared to only 0.05% of retrospectively reported cases (Bolen, 2003). This 880-fold increase has been attributed to the practice of labeling mothers as offenders when they are believed to have allowed abuse to occur, despite not directly perpetrating the abuse. The consequences of guilt by omission can be detrimental, including custody loss and criminal prosecution. Against a backdrop of emotional distress, competing allegiances, scarce resources, and intersecting oppressions, the life-altering tasks involved in maternal protection commonly go unnoticed by CPS (Davies & Krane, 1996). Following the abuse of their child, mothers can experience traumatic stress and other mental health sequelae, in addition to social and material losses (Elliott & Carnes, 2001). Notwithstanding these adversities, most act in their child's best interest postdisclosure. Although ambivalence is recognized as a normative early reaction to CSA, the majority of mothers respond supportively (Bolen, 2002), with some evidence of association to improved child outcomes (Bolen & Gergely, 2015). Maternal narratives, however, uncover pejorative and punitive interactions with service providers in the aftermath of CSA, potentially exacerbating crises and diminishing help-seeking (Alaggia, 2002; Plummer & Eastin, 2007a, 2007b). Encounters with blame in the face of overwhelming expectations can impede maternal capacities for support and protection and, thus, compromise child safety and recovery.

Formulating CSA as a consequence of maternal inadequacies wrongly, albeit effectively, shifts the locus of accountability away from the individual committing the offense and the power asymmetries endemic to our society that sanction its existence. Although it has garnered attention in the intimate partner violence (IPV) literature (Fugate, 2001; Kopels & Sheridan, 2002; Magen, 1999; Strega, 2009; Strega et al., 2013), the current state of empirical knowledge on FTP in the context of CSA is notably deficient despite its perilous effects on women and children. My primary objective in this study, therefore, was to shed light on how gendered relations of power operate to construct and reinforce discursive notions of blame and FTP in child welfare system responses to the sexual abuse of children by way of policy and practice texts. In the tradition of critical social research, the goal of this work was to expose and destabilize dominant ideologies of gender, motherhood, and risk, and to build a persuasive case for social and institutional reform grounded in an alternative discourse with sounder explanatory power.

## Method

Partial findings of a doctoral dissertation approved by the University of Toronto Health Sciences Research Ethics Board are reported (Azzopardi, 2015). Feminist critical discourse analysis (CDA) was applied to investigate discursive dimensions of blame and FTP in child welfare policies and practices related to CSA. With a progressive social advocatory impetus, CDA is a transdisciplinary, problem-oriented, multimodal approach to the study of discourse (Fairclough, 2010; van Dijk, 1993; Wodak & Meyer, 2009). Understood as social practices that are socially conditioned and socially constitutive, discourses are semiotic ways of construing aspects of the world, including language use in speech and writing, with significant ideological effects that reinforce or resist particular relations of power. A main objective of CDA is to uncover and remedy social wrongs with the knowledge generated through the critique of text in context. This investigation's attention to gender called for an explicit feminist discourse praxis (Lazar, 2007), especially relevant in current time and space where unequal power dynamics and social arrangements based on gender are increasingly subtle yet equally toxic. Inherently centered on subjective judgments of a just society and rejecting the possibility of wholly value-free research, this study was engaged from a transparent sociopolitical stance with a social change agenda.

### Data Collection

Child welfare is a highly verbal field with heavy reliance on policies and procedures, whereby oral interactions of great consequence are preserved through writing. As such, the vast availability of textual data makes this rich ground for CDA. The scope of this study was restricted to the contemporary state of evolving child welfare discourse in Ontario, Canada. Its sample was extracted from a network of existing child welfare texts from interrelated genres—law, policy, practice tools, case files, and court documents. Each was purposively selected on a principled basis as semiotic points of entry into the object of study (Fairclough, 2010). The first phase of data collection entailed locating documents that were influential in shaping current child welfare services and satisfied the following criteria: (a) statute, policy, or practice standard/tool regulating or instructing child protection work; (b) relevant to the investigation of CSA and FTP dispositions; and (c) written in English and publicly accessible. To this end, my preexisting knowledge of the field was applied, local authorities in child welfare administration and quality assurance were consulted, and online searches were conducted on child welfare agency and provincial government websites using various keywords combined with the Boolean operator “AND” (e.g., child welfare, child protection, law, standards, service delivery, Ontario). The following six documents were identified: (a) *Child and Family Services Act* (CFSA, R.S.O. 1990)—provincial legislation governing child welfare services in Ontario; (b) *Child Welfare Transformation* (Ministry of Children and Youth Services [MCYS], 2005)—strategic plan for transforming Ontario's child welfare service delivery model; (c) *Child Protection Standards in Ontario* (MCYS, 2007)—policies and practices establishing the mandatory framework within which child welfare services are delivered; (d) *Ontario Child Protection Tools Manual* (MCYS, 2007)—instruments for screening and assessing child protection concerns; (e) *Ontario Child Welfare Eligibility Spectrum* (Ontario Association of Children's Aid Societies, 2006)—supplementary decision-making tool for service eligibility and severity of harm; and (f) *Ontario Child Protection Tools Manual and Child Welfare Eligibility Spectrum Policy Directive* (MCYS, 2007)—policy directive mandating the application of screening and assessment tools. These documents totaled ∼480 pages of text.

The next phase of data collection involved acquiring a retrospective sample of CPS case files from one agency situated in an urban region of Ontario. With institutional consent, database searches of demographic data, service eligibility and severity of harm coding matrices, and verification dispositions were conducted to locate files that satisfied the following criteria: (a) case was referred within the past five years, ensuring services were informed by current policies and practices; (b) primary reason for referral was suspected sexual abuse of a child under 16 years; (c) primary caregiver was identified as the mother and not suspected to have perpetrated the CSA; and (d) investigation was complete and protection application was filed with the court in relation to verified concerns of maternal capacity/FTP in the context of verified CSA. File recordings included referral details, investigation reports, caregiver profiles, safety and risk assessments, strengths and needs assessments, dispositions and service plans, case and supervision notes, and court documents (affidavits, protection applications, status reviews, temporary and final orders, plans of care, statements of agreed facts). With data collected and analyzed simultaneously, a deeper reading of files meeting inclusion criteria was conducted to identify cases containing strong manifestations of the constructs of interest. This culminated in the purposive selection of three files consisting of ∼870 pages of text. Sample completeness was reached when emerging data were sufficient for principled study, satisfied the research objective, and supported a persuasive case for change (Wodak & Meyer, 2009).

In the final phase of data collection, negative case sampling was applied to purposively select one CPS case file that met all of the above criteria but with no secondary protection concerns related to maternal protective capacity. This single file, consisting of ∼40 pages of text, was included for comparative purposes. It extended analyses by demonstrating areas of convergence and divergence in discourse in the presence and absence of FTP. Together, these 10 sources of textual data, totaling ~1,390 pages of text, offered a comprehensive picture of the current state of child welfare discourse as it relates to semiotic representations of gendered attributions of blame and FTP in cases of CSA. Every step in the child welfare process was observable and accountable through this systemically linked network of texts.

### Data Analysis

Under the umbrella of Lazar’s (2007) feminist CDA paradigm, this study drew from Fairclough's (2003, 2010) dialectical-relational framework for critically analyzing discourse. This approach allowed for concurrent points of analytic entry into three interrelated elements of discourse, where microanalysis of written text was linked to macroanalysis of social context. The first dimension, discourse-as-text, involved systematic descriptive analysis of linguistic features of concrete instances of discourse. The underlying assumption here being language, as a social practice, is never arbitrary or neutral but serves ideological functions, though not always transparent. The second dimension, discourse-as-discursive practice, involved interpretive analysis of practices and processes that connect text to the social context in which it was produced and received. This interceding position was occupied by intertextuality and interdiscursivity. The third dimension, discourse-as-social-practice, involved social explanatory analysis of sociocultural conditions and ideological effects in which discourses have taken shape.

The analytic process was nonsequential and involved simultaneous description (text analysis), interpretation (process analysis), and explanation (social analysis). Elements of feminist CDA were infused, including the principle of gender relationality (Lazar, 2007). In-depth analyses concentrated on sections of text directly relevant to the study focus. Electronic searches of keywords embedded within large texts helped to identify concepts of interest and perform frequency counts. A sociodemographic data collection form and data analysis coding structure aided in the systematic organization of data into manageable units, and reflective memos were used to track analytic insights. Documents were initially engaged undiscerningly as a whole; meaning, each was read from beginning to end *with* the text before a disruptive reading *against* the text. This abductive, iterative process involved cyclic readings, with attention paid to both manifest and latent content, and movement between theory and data. Each text was analyzed for emerging patterns of complementary, contradictory, and competing discourses, as well as institutional and cultural conditions that gave rise to the text.

## Results

Descriptive, interpretive, and explanatory analyses are threaded through a broader discussion of the social terrain that influenced child welfare text production and reception. Following general observations of the documents and demographics, dominant discourses emerging from the data are reported thematically. With no pretense of absolute neutrality, expressions of discourse are evidenced by italicized quotes extracted from raw texts (with identifiers omitted). While acknowledging and affirming gender nonbinary persons and the importance of gender inclusive language, gendered nouns and pronouns (e.g., women-men, she-he) are used with intention to accurately capture the contents of the dataset and gender ideology reflected therein.

### Language of Child Welfare Texts in Context

Instrumental in organizing, administering, and standardizing child protection work, the six provincial child welfare documents reviewed were authored by a government/legal authority or professional group, each selectively drawing on the expertise and interests of stakeholders and advisors. Indicative of the power held by these institutions, the formality and instructional purpose of the texts reflected a mostly authoritarian voice. Entrenched in the tradition of law, the CFSA was imbued with directive speech acts issuing performative CPS functions fortifying its power. Although the Child Protection Standards explicitly stated that authoritative language was avoided to “reflect a shift in the culture or philosophy of service provision toward more collaborative, strengths-based approaches,” it consisted of prescriptive statements and, as ministry-enforced policies that articulate expectations for service delivery, was inherently imperious. There was ample evidence of intertextuality in each document, with references to and reliance on the content of other hierarchically ordered texts, highlighting their co-construction and function in relation to each other.

The CPS case files reflected an array of voices filtered through the lenses of professionals in ranked and specialized roles within the confines of institutional structure. The language practices of workers recontextualized those of policy and practice tools, resulting in a hybridization of discourses, styles, and genres. Subject to close scrutiny and court subpoena, the standardized format of file documentation provided a sense of completeness and impartiality. The simplified version of “facts” that workers opted to record (and omit) were selected in accordance with organizational requirements and influenced by personal schemas. Linguistic style ranged from informal conversational tone to formal legal jargon, all more or less authoritative in nature. Forms presented to the court mirrored file texts. Recordings were highly repetitive, thereby qualifying and reinforcing particular impressions, findings, and labels. Although necessary for building a case for mandated intervention (or termination), this effectively reproduced certain discourses and suppressed others. All notes of maternal actions and statements were written from another's perspective and, therefore, were limited to what was discernable. Some were objectively logged with concrete descriptions of observed behaviors and verbatim dialogue, whereas others appeared more subjective and value-laden. With little power over the words that made it to the page, mothers, for the most part, were the passive subjects of institutional talk and text practices.

### Child Protection Case File Demographics

The sample of four CPS case files involved substantiated sexual abuse of girls (11–14 years) by a biological father or stepfather in a cohabiting relationship with the mother at the time. A joint or parallel investigation was conducted by CPS and police in each case, resulting in criminal charges of two of the four men. None of the women was criminally charged. In all but the negative case, the children were removed from maternal care due to perceived failures in protection. All but one were subsequently returned with years-long court-ordered supervision by CPS. The primary reason for involvement was verified CSA by a father figure, though the mothers, three of whom ultimately became sole-support parents, were the main target of intervention. Each family identified as a racial or ethnic minority and, of the three FTP cases, all were classified as low-income. There was no documentation to confirm maternal mental health or substance abuse problems; however, all had personal histories of IPV and trauma.

Worker demographics were not available. General profiling data from other sources will augment the context of CPS responses. Child welfare is a feminized profession marked by a gendered occupational discourse (Scourfield, 2006). Although males occupy more space in upper management, the vast majority of the frontline workforce is female, white, and English-speaking (Public Health Agency of Canada, 2010). Personal attributes and experiences color worldviews and constructions of parenting. Operating from a dominant Eurocentric orientation, child welfare systems have historically had harmful relations with Indigenous and Black communities, with ongoing systemic discrimination underlying referral and outcome disparities (Ontario Human Rights Commission, 2018). Taken together, worker and mother profiles represented individuals who were multiply positioned in the world; each dyad inevitably shaped by layered power imbalances by virtue of social category and professional authority.

### Intersecting Discourses of Gender, Motherhood, and Risk

#### To Protect and Support? Determining Children's Best Interests via Maternal Capacity

Child welfare trends have been likened to a pendulum that swings between crisis-driven extremes embodying competing discourses of state intervention and family preservation (Dumbrill, 2006). The promise of change in Ontario came with system-wide reform known as the child welfare transformation agenda, intended to balance the pendulum by enabling child protection and family support to coexist. Using tactical language signaling a commitment to progressive change, the agenda aimed to transform the service model to one that embraced collaboration, strengths, and customized responses. Primacy of the child nonetheless remained codified in the CFSA, which underscores the paramount purpose of the Act as to “promote the best interests, protection and well being of children.” Resting on notions of childhood as a vulnerable stage of development rendering children in need of special rights and protections, an acritical reading might take the child focus as a given. The legal objective is after all to uphold their welfare. The language used to institute paramountcy, however, sets in motion a strongly worded child-centered discourse that positions their safeguarding above all else, rivalling against woman-, parent-, or family-centered discourse.

The *best interests of the child* is a rights-based governing principle and overriding consideration in protection dispositions (Walter et al., 1995). Despite emblematic appeal, best interest interpretations are ambiguous. The adjective *best* implies a hierarchy of interests yet the statutory list of criteria is not rank-ordered, allowing for imposition of personal ethics and agendas on a poorly operationalized but consequential concept. Speculating what might be best for any one child is an arbitrary process of highly individualized choices between alternatives without necessitating rational reasoning (Skivenes, 2010). This is problematic when discrete categories compete, and when dominant worldviews and preferences of privileged decision-makers conflict with those of oppressed women and children, thus weakening consistency and fairness. Sometimes at odds with the needs of mothers, this case file analysis revealed that children's best interests were evaluated almost exclusively against maternal capacities to protect and resultant perceptions of risk, with little appraisal of other important factors. In each disposition, risk of CSA superseded other considerations, including continuity of care, risk of emotional harm, and personal views and wishes. This reflects conventional protection praxis that weights physical over nonphysical harms (English et al., 2015) and undermines the agency of women and children. Tensions in the dual child welfare mandate are cemented by the paramountcy of child-centric best interests. In all but the negative case, helping discourse was mostly eclipsed by protection discourse. With protection enacted as safeguarding children from CSA via maternal FTP through intrusive measures marked by power disparities, opportunities for empathic engagement, support, and guidance were missed.

#### Masking Mother-Centrism: The Pretense of Gender-Neutral Language

In pursuit of inclusivity, there has been movement toward gender-neutral vocabulary in child welfare. Given the significance of naming choices, an important observation in this study was the systematic adoption of ungendered terminology in the policy and practice documents reviewed. As presented in Table 1, the texts showed a proclivity toward using the genderless common nouns *parent* and *caregiver* in place of the traditionally genderful common nouns *mother* and *father*. Gender ambiguity in lexicon conflates mothering and fathering into a homogenous category, implying that texts do not have a stable addressee. Upon closer examination, however, it was apparent they were partial toward the role of mother. For example, *parent/caregiver* was defined in the CFSA and Eligibility Spectrum as the *mother* first in a given list of individuals, the sequencing of which reinforces primacy. Use of singular tense explicitly addresses one individual, thus discounting increasingly popular discourses of shared parenting and involved fathering. In practice, this translates to no mandated engagement with secondary caregivers without equal childcare or legal responsibilities. In the neoliberal era of managerialism driven by cost-effectiveness and efficiencies, gender-neutral language provides an opportunity for shortcuts in practice (Brown et al., 2009). Discourses of caregiving are steeped in motherhood imagery and synonymous with mothering in child welfare work. The upshot of this naming strategy is the obfuscation of the gendered state of parenting and protecting. In effect, institutionally and culturally engrained, gender-based ways of thinking and doing may be more powerful than the seemingly gender-blind language of laws and policies.

**Table 1.** Frequency of References to Gender-Specific and Gender-Neutral Caregiver, Risk, and Failure to Protect in Child Welfare Texts.

Child welfare document	Mother^a^	Father^a^	Parent^a^	Caregiver^a^	Guardian^a^	Person having charge	Risk^a^	Fail^a^ to protect^a^
Child and Family Services Act (125pp)	2	2	258	0	0	9	22	9
Child Welfare Transformation Strategic Plan (27pp)	0	0	17	1	0	0	19	0
Child Protection Standards (92pp)	4	0	83	6^b^	4	0	182	4
Child Protection Tools Manual (120pp)	5	3	399	397^b^	5	0	204	6
Child Welfare Eligibility Spectrum (113pp)	4	4	128	435^b^	6	81	210	31^c^
Policy Directive (3pp)	0	0	0	0	0	0	2	0

^a^ Plus suffixes.

^b^ Excluding references to *community caregiver*.

^c^ Excluding references to comparable phrases (e.g., *failure to act, failure to care for, failure to provide for, failure to supervise, neglect in protecting, inability to protect, does not act to protect*).

Gender biases were also apparent in the naming choices of workers. Women were more likely to be referred to with the common nouns *mom* or *mother,* whereas men were more likely to be referred to with proper nouns such as their first or last name and title (e.g., statements like “I don't think mom can protect the child from Mr. [X]” were commonplace). Naming a woman solely in terms of her maternal role detaches her from her other equally important identities, and narrowly defines her and what we expect of her according to connotations of motherhood. Conversely, addressing an abuser by his given name preceded by mister fortifies his individual identity as a dominant male, conceals his paternal role and responsibility, and bestows a sign of respect not afforded to women. Predisposed naming choices and their associated social ranking created a sense of opposition in the files between deidentified mothers and identified men.

#### Obliged Mothers, Discretionary Fathers, and Invisible Offenders: Deflecting the Gaze

Notwithstanding the widespread use of gender-neutral language, gendered practice was evident in the case files through all stages of service delivery. Recordings were disproportionately occupied by maternal references. At the time of entry, cases were opened under the mother's name by default. This established early concentration on mothers, with an underlying implication of culpability and concurrent invisibility of fathers. Regardless of whether the men involved were assets or risks, attention remained relatively fixed on women, whose roles in care and protection were deemed fundamental obligations. Mothers were labeled as the primary caregiver for every safety and risk assessment. In one case, the nonoffending father was mostly absent from his child's life and thus constructed as irrelevant in the texts through silences. In two cases, the nonoffending fathers were present but the nature and extent of their involvement were discretionary based on quality of maternal care. In other words, these fathers were engaged by CPS, at least initially, because circumstances demanded them to be. Contact with these men would likely have been deemed unnecessary had the women been adequately protective. Both fathers assumed temporary care of their child during the investigation, filling a nontraditional gender script. Their role as primary caregiver was short-lived and their ongoing responsibilities in protection were minimal, yet their brief contributions to basic care were at times portrayed as heroic actions deserving of praise. Although offering evidence of discursive resistance to mother-centrism, the following quote extracted from a case note illustrates one worker's compassionate engagement and commendation of a father with a long history of absenteeism and violence:

I acknowledged that he approached his daughter from a place of wanting to protect her, loves and cares for her, and never did I question his love for his daughter. … I am fighting with him for his relationship with his daughter. … He seemed to understand where I was coming from.

This text conveyed a sense of mutual empathy and validation, something lacking from most logged accounts of worker–mother interactions. The gradual textual invisibility of fathers coincided with ever-present maternal visibility.

Another emerging theme was the dearth of documented contact between workers and offenders, despite them being the main source of risk. Attempts to communicate directly with them regarding case planning or access visits were generally met with avoidant or resistant behaviors. Mothers were consequently positioned as gatekeepers (e.g., directions such as “I asked [mother] to let [stepfather] know we need to talk and he needs to provide all the information regarding conditions and programs he attended” were regular). There was no suggestion of maternal obstruction to access. To the contrary, they took steps to facilitate contact, even if it came with risks to their own safety. A related observation was the quantity of information recorded about men, which was proportionally less compared to women. This was especially evident for those without a biological or legal relationship to the child. The paternal details that were documented were based mostly on maternal report, discounting the need for direct CPS interaction with fathers. This may signal fear or unpreparedness to work with men, particularly violent men who pose a threat (Maxwell et al., 2012a). The consequences of gendered child welfare practices are amplified expectations and scrutiny of women, erosion of men's accountability, and maintenance of patriarchal social order.

#### Entrenching Risk Intolerance: Social Relations of Risk

Rapid detection and removal of risk are foremost objectives in child protection work. To illustrate the materialization and institutionalization of risk discourse, Table 1 reports the frequency with which the term *risk* appeared throughout the documents. Defined and applied inconsistently, there were 639 instances of *risk* in 480 pages of text, remarkably so in the practice standards and instruments. The power effects of discourse lie in recurring language, the continuous repetition of which solidifies and sustains certain ideas. Risk is a salient concept in the current study because judgments of CSA likelihood secondary to FTP are founded in evaluations of risk. Although all risk connotes danger, the threat of sexual abuse triggers a panic state more so than other forms of maltreatment. In CSA policy and practice, this translates to no or very low tolerance for risk (Carlton & Krane, 2013).

Risk is an ideologically loaded construct with meanings contingent on social contexts, moral functions, and political agendas (Swift & Callahan, 2009). Child protection agencies occupy the space between state and individual. As agents of the state, workers exert their authority with governing technologies such as actuarial models of risk assessment. This surveillance occurs in the climate of neoliberal governmentality (Foucault, 1991), which transfers risk and responsibility onto the rational actor, sanctioning state retreat from social welfare obligations. Conflated with fear and liability, the construction of risk discourse is a mechanism for resource allocation in the face of meager budgets. Empirical measurement of risk conveys objectivity, with science positioned atop the knowledge hierarchy. Risk appraisal using validated tools remains highly variable, however (Regehr et al., 2010). Largely perception-based and devoid of structural analysis, binary risk criteria can reflect oppressive relations of gender, race, and class while concealing conditions of mothering (Krane & Davies, 2000). In the files reviewed, CPS dispositions appeared to be based predominantly on subjective judgments of maternal capacity to eradicate risk rather than calculated risk. All risk assessments scored ratings of moderate or high risk, yet the negative case had a drastically different outcome than the FTP cases (case closed vs. court-ordered supervision or state care). Computations were commonly increased at worker discretion based on maternal characteristics. Discretionary ratings allow for case complexities and practice wisdom to be considered. They also open a window through which personal biases can enter.

#### Targets of Blame and Agents of Change: Maternal Embodiment of Risk

There are multilevel, static and dynamic, biopsychosocial correlates of CSA (Assink et al., 2019; Levenson & Morin, 2005). Formulating a sound finding of risk requires integrating information from all levels of ecology, the complexity of which defies simplified checklists and fleeting timelines. Despite existing across the ecosystem, the construct of risk in each file was circumscribed almost exclusively to maternal (in)action, (un)knowing, and (ill)intent—past, present, and future. Generally disregarding perpetrator and environmental risk factors, all risk assessments, care plans, and court petitions narrowly concentrated on the failures and faculties of mothers to protect. Immediate CSA risk was eliminated by removing the child from maternal care. Responsibility for remedying the risk was then placed with the identified source of threat, the mother. In the cases with reunification, ongoing risk was mitigated by surveilling and regulating maternal behaviors and attitudes in an effort to monitor and control offender access, with escalating threats of more intrusive measures.

Future risk was conceptualized primarily as the likelihood of CSA recurrence consequent to maternal inability or unwillingness to prevent contact with the offender (risk to physical safety) and secondarily as an unsupportive maternal response (risk to emotional well-being). The latter, based on presupposed clinical and empirical knowledge, garnered less attention. As shown in the following CPS recording of a home visit, CSA risk was contingent on maternal willingness to protect:

We discussed about [stepfather] and how she is willing to protect her kids from other incident. [Mother] indicated if they reunite and something like that happen again, she will ask him to move. I told her, she is placing herself in the situation that this could happen again. The thing is how we can prevent that, and not allowing further incidents. [Mother] was confused and indicated she needs some time to think about this. … I told her we will have this conversation again in 3 months, and I hope she will present a plan to me regarding her children and how she is going to protect them. She agreed.

[Three months later]

I reminded her that 3 months ago we talked about what is her pan [sic] to protect her children from [stepfather] when rereleased [from jail]. She said she will watch them closely. She will talk to her mother to watch the kids while she is working. … We discussed about preventing versus acting afterwards. I told mom I need to hear a plan to prevent the children from being harmed, I need a plan where mom will protect the kids before any incident occurs.

Here and elsewhere in the files, CSA was represented passively and without agency; meaning it was referenced with nondescript language such as *incident,* without identifying the act of abuse or person responsible for committing the abuse. In contrast, active voice was used to unequivocally link the act of protecting (or failing to protect) to the mother. The text reinforced maternal responsibility for eradicating risk (and fault for failing) while suppressing paternal accountability for abusing. Use of the plural pronoun *we* in the statement “how we can prevent further incidents” implied risk management would be collaborative. This was preceded and followed by directive speech acts using the singular pronoun *she,* locating sole agency with the mother in the absence of contextual considerations and help to execute a realistic protection plan. The mother's efforts and strategies were met with dissatisfaction. Should abuse recur, she was avowed to be “placing herself in the situation,” thereby preordaining maternal blame, deflecting responsibility from the offender, and curtailing liability of the worker, agency, and system.

In the following citation from a court document requesting a CPS supervision order, grounds for risk remained firmly situated in maternal behavior, knowledge, and attitude:

There are reasonable grounds to believe that the children are at risk, due to the following concerns:

1. [Mother] was not able to protect her daughter from her stepfather and blamed [child] for the incident.
2. [Mother] claimed that she did not know about [stepfather's] behavior.
3. [Mother] has a history of excusing [stepfather] for all of his past criminal charges.
4. [Mother] did not follow through with recommendation that any access between [stepfather] and her children be fully supervised…

In each risk claim, the mother was positioned as grammatical agent, wherein she was actively identified as the cause or initiator of events. Linguistic modality, voice, agency, and rhetoric were used to make a compelling argument to the court demonstrating current risk based on past maternal failures while unambiguously backgrounding perpetrator risk. Grounded in silent discourses of proper mothering, these reasoning strategies appealed to emotion and ethics. The contention of unsupportive maternal response to the child indeed posed a quandary, given its potentially harmful impact. Underlying assertions of risk were insinuations of bad mothering that rationalized blame and legal consequence. Between the two quotes, risk shifted from willingness to protect from future abuse to ability to protect from past abuse. The adjective *willing* presumes available options and a degree of readiness; thus, unwillingness to protect is a matter of personal choice or unsatisfactory efforts. The adjective *able* implies the possession of power, opportunity, and means to act; thus, inability to protect is a matter of incapacity, regardless of intent. The nominalized noun *ability* turns the construct into a tangible entity that, by definition, should account for barriers to protection, but nonetheless obscures case complexities and inequities.

Discourses of risk gain their power by drawing from dominant ideologies of individualism, morality, and blame (Webb, 2006). Mothers were constructed as the embodiment of risk and target of blame for failing to perform their protective function up to par, while paradoxically appointed as the principal agent of change. The texts spoke in a tone of relative certainty about issues fraught with uncertainty. The ambiguity of risk was masked by technical language describing forensic procedures and numeric scores justifying courses of action. Through these textually mediated processes, particular versions of knowledge were legitimized as fact at the exclusion of others. Most evaluations of risky mothering had as their foundation subjective interpretations reflecting a moral stance and fear-based reasoning, rather than an objective and rigorous appraisal of the chance of harm, accentuating the ideologic over the pragmatic.

#### FTP in Policy and Practice: Institutionalizing Mother-Blame

FTP as legal grounds for protection gets translated through child welfare policies and practice standards and operationalized through assessment instruments and everyday practice. To illustrate the extent of entrenchment, Table 1 presents the frequency of the phrase *fail to protect* in the documents. With 50 recurring instances (excluding comparable terms), the power effects of FTP discourse in child welfare are evident. Solidified in the CFSA, a child is deemed in need of protection when:

37 (2) (c) The child has been sexually molested or sexually exploited, including by child pornography, by the person having charge of the child or by another person where the person having charge of the child knows or should know of the possibility of sexual molestation or sexual exploitation and fails to protect the child; (d) there is a risk that the child is likely to be sexually molested or sexually exploited as described in clause (c).

A surface-level reading of this legislation might begin and end with its taken-for-granted assertion of caregiver duty to protect. A closer interdiscursive examination, however, unveiled evidence of interlaced discourses of risk and blame that rely on and reproduce gender-based ideologies of motherhood to fortify particular relations of power. To augment this analysis, units of information were broken down into stanza form:

1. the person
2. having charge of the child
3. knows or should know
4. of the possibility of sexual molestation or sexual exploitation
5. and fails to protect the child

The common noun *person* (1) is used to actively identify the gender-neutral subject of responsibility, though it is linked to the clause “having charge of the child” (2), implying a duty of care toward the child at the time of abuse or risk of abuse. Because most children are cared for primarily by mothers and other female caretakers, there is an uneven effect on women by virtue of group membership. Use of *person* in singular tense individualizes responsibility for protection and averts liability from secondary caregivers and broader society. In the next clause, “knows or should know” (3), the verb *knows* infers requisite knowledge of the possibility of abuse while the added modal auxiliary *should* signals a subjective mental element based on an expectation of intrinsic ability to know or predict risk. Informed by hidden discourses of motherhood, this assumption conjures the notion of maternal instinct, wherein all good mothers should intuitively know when their child is in danger. Fathers are not presumed to possess this natural feminine ability and thus are not usually held to this standard to the same degree. The following clause, “of the possibility of sexual molestation or sexual exploitation” (4), lies on the supposition of predictability and connotations of danger. Here, responsibility for protection expands from abuse to risk of abuse, securely locating FTP as a marker of risk. The final clause, “and fails to protect the child” (5), rests on four assumptions: (a) caregiver had a duty to protect the child in their care from avoidable harm and risk of harm, (b) nature of harm and circumstances leading to it were foreseeable and controllable, (c) caregiver failed to take reasonable measures to recognize and prevent or stop the harm, and (d) caregiver was therefore responsible for the resultant harm.

The specific actions or inactions required to constitute FTP are not demarcated in this broad statute, leaving the opportunity for discretionary judgment and gender bias to seep in. Couched in gender-neutral language, *fail to protect* appeared most frequently in the Eligibility Spectrum as intertextual references to the CFSA. To fall under the intervention line, caregivers are essentially expected to accurately predict CSA or risk of CSA, take rapid and reasonable steps to protect, *and* be successful in their efforts—resonating ideology of the omnipotent mother. These sentiments manifested in file recordings, where FTP was formulated without attention to overlapping but discrete dimensions of maternal responsiveness and multilevel variables that impede protective capacities. Disbelief and ambivalence were conflated with unwillingness or inability to support and protect, contrary to research challenging construct synonymity (Bolen & Lamb, 2007). Expectations of steadfast belief persisted in spite of uncertainties. Arguably, there were plausible reasons for doubt as mothers grappled with the allegations, including adamant denials and coercion by the men they trusted, tentative disclosures and recantations by the children, and conflicting forensic outcomes in child welfare and legal systems.

#### Manufacturing the Power to Protect: Gendered Ideologies of Motherhood

Mothering discourse is the hybridization of parenting discourse and gendering discourse. FTP doctrine gains its power by drawing on and activating essentialist constructions of motherhood to render women assailable for the sexual offenses of men. Here we return to a critical analysis of the four suppositions inherent in FTP discourse, which together build a generalizable argument schema. The first is that mothers, irrespective of circumstance and preference, have a social, moral, and legal duty to protect their child from avoidable harm. Rooted in the institution of motherhood (Rich, 1976), liabilities of childcare stem from a gendered division of parenting roles based on the so-called natural order of things, with rules of maternal conduct grounded in patriarchal, ethnocentric, heteronormative, and middle-class values. There was evidence in the case files to suggest the good–bad mother binary infiltrated protection decisions, expectations, and interactions. In their exchanges with workers, women were judged against idealized standards of mothering, reminded of their maternal deficiencies, and instructed on how to satisfactorily perform their jobs as good mothers. FTP judgments hinged on familiar but antiquated narratives of the all-sacrificing, all-knowing, all-powerful mother (Epstein, 1999; Johnson & Sullivan, 2008). The all-sacrificing mother naturally, lovingly, and selflessly puts the needs and interests of her child above those of her own, regardless of obstacles and consequences. The all-knowing mother has an instinctive capacity to sense when her child's safety is in harm's way and the intuitive wisdom to know what to do about it. The all-powerful mother possesses the unconstrained ability, by virtue of physiology, to protect the well-being of her child, notwithstanding social location. Evaluations against these maternal stereotypes were prominent in the files:

The all-sacrificing mother:

There is uncertainty in her disposition to be able to put the needs of the children first.

We need her to be able to show that she can protect her daughter and keep her safe and not put her relationship before her daughter's safety.

[Counsel] spoke to the judge that mother could choose her husband over her child and there could be implications.

The all-knowing mother:

When faced by [worker], mother denied knowing the situation. She indicated that she didn't know anything as she was working that day. I told her that her husband was arrested and she didn't know why? She insisted she wasn't home.

Mother was informed by [worker] that she was not able to protect [child] from [stepfather]. More than that, she blamed her daughter. Mother indicated that she is able to protect her children, that everything happened while she was not at home, and that she never knew.

Mother was tearful during visit, stating that she did not want children to be removed. She explained that she was working full time, she was unaware of these concerns.

The all-powerful mother:

Mother and maternal grandmother who live at the same place as [stepfather] were unable to prevent this incident and denied being aware of father's actions. In any case, all the incidents happened while mgm and mother were home, and they couldn't prevent it. Then I don’t think mom or mgm can protect the children from [stepfather].

I told her she, as a mother, must protect her daughter from such embarrassing situation. Now her role as a mother is to support her, being there when she needs her the most, not blaming her. I asked her to put herself one moment in her daughter shoes. … How would she feel? [Mother] started crying.

I told them, they have 2 options, [stepfather] leave right now or I will apprehend the children. … I informed mother that I'll proceed with a protection application at the court and will ask for a supervision order. Then the children will be under the supervision of [CPS]. As mom was in tears when I was leaving, I told her she needs to be strong because she was about to give birth, and that she needs to take care of her children.

[Mother] shall ensure that [stepfather] does not reside at the family home. [Mother] shall ensure that [stepfather] has no access to the child. [Mother] shall ensure that [stepfather] has no access to the [sibling]. [Mother] shall advise [CPS] forthwith if [stepfather] attempts to have access with [child or sibling] in contravention of the access orders made in these proceedings.

Failing mother's ability to protect her children, the children would be apprehended.

These texts located themselves in contradictory positions, suggesting that women were both powerful in relation to men and powerless in relation to the state. Although not to abate the ethical dilemma of seemingly unsupportive maternal responses and their adverse effect on child well-being, gravitating to dominant discourses of mothering functioned to intensify blame and shame when mothers failed to live up to idealized archetypes. Case information that challenged fixed notions was met with resistance. For example, contesting the myth of maternal instinct or intuition, women's denials of knowing about CSA prior to disclosure were received with skepticism or met with insinuations of turning a blind eye, elicited intimations of blame for being absent from the home, or were disputed with accusations of missed warning signs and failure to decode subtle clues. Because good mothers should have and would have known.

Conversely, the mother in the negative case measured up to the good mother paradigm:

Mother is protective of [child], is living with a friend in [city] and will not give access to the father. She has been co-operative with the [police]. … Mother has full custody of the children and will be following through with [CPS] referral to [counselling agency] so that [child] can get therapy. Mom believes her daughter and stated she will do whatever she needs to protect her. Mother is supportive and protective of her daughter and involved in supportive services in the community. File to close.

Here, proper mothering entailed instantaneous and unwavering belief, the immediate termination of marriage, full cooperation with the investigation, physical protection and emotional support of the child, and commitment to “do whatever she needs to” to ensure ongoing protection. Unlike those in the FTP cases, this mother was willing and able to carry out the challenging tasks required for protection and had the social support and finances to do so promptly and effectively. File closure was legitimized by discourses of maternal self-sacrifice and compliance.

The second assumption underlying FTP is that the nature of harm was not inevitable. In other words, it could have been predicted and prevented via acts within human agency. Through the linguistic process of nominalization, the verb *fail* is transformed into a concrete entity with the noun *failure,* making it appear tangible and inarguable. Ideologically, this functions to present something contestable as incontestable. A core implication of the word *failure* is that circumstances were controllable; there was an opportunity to not fail (Magen, 1999). The word *protect* infers possession of power. Fulfilling the duty to protect therefore requires both opportunity and power to act. CSA is a “man-made” phenomenon; its dynamics of secrecy and shame typically leave few or no detectable signs (Gilgun & Anderson, 2013), as seemed to be the case in the files reviewed. Hence, maternal failure may have been inevitable. When there was opportunity to act, women were erroneously assumed to be sufficiently empowered to control the actions of men.

The third assumption implies the caregiver failed to take reasonable measures to recognize and prevent or stop the harm. Standards of reasonableness are abstractly understood as what a rational person using sound judgment would or should know and do under similar circumstances. As this analysis showed, however, reasonable protective measures were poorly defined and operationalized in policy and practice texts. No consideration was given to contexts under which it might be reasonable to not act. Evidence emerged from the files to suggest that expectations were not realistically grounded in the hardships and risks faced by individual mothers. Universal application may have operated unfairly against racialized and otherwise marginalized women, where the conditions required to adequately fulfill the protection role were generally unacknowledged. Together, these assumptions advance a causal link between maternal FTP and the resultant harm or risk of harm to the child. The problem of CSA therefore becomes largely defined in terms of women's acts of omission over men's acts of commission.

#### Decontextualizing Women: Harsh Realities of Mothering and Protecting

Balancing the safety and well-being of children and mothers is a challenging feat in child welfare. Throughout the case files, there were glimpses of the bleak contexts of women's lives, personified by acute and chronic crises, interpersonal violence and trauma, parenting stress and loss, and financial insecurity, with hints of resiliency. For the most part, however, their day-to-day experiences went critically unexamined and virtually disappeared through the documentation process. Again echoing competing discourses of supporting and protecting, there were few connections in the texts between maternal adversities and capacity to protect, with expectations and consequences persisting regardless of circumstance.

Despite high prevalence of co-occurring CSA and IPV (Holt et al., 2008), there was no explicit link in the policy or practice documents between men's violence against women and women's ability to protect children from sexual abuse by violent men. Although some mothers can parent effectively through violence, decision-making and competency can be compromised by trauma, fear, and perpetrator strategies to undermine women's mothering, estrange support networks, and control resources (Lapierre, 2010). There was a documented history of IPV in each file, though references were vague and overshadowed by CSA risk. A theme cutting across all cases was the imposed condition that mothers instantly and permanently separate from CSA offenders should they want to retain or regain child custody. In this zero-tolerance approach, risks to women's safety were unheeded through textual silences. The staunch expectation to immediately sever ties is based on the erroneous assumptions that women are liable and leaving is a viable option and effective solution, with scant regard for the heightened threat of injury and death postseparation (Campbell, 2007). Risks intensify when leaving is not executed planfully, as held true in the midst of these CSA disclosure crises. In the context of child-centric notions of best interests and CSA risk intolerance, complex processes were reduced to a maternal “choice” between partner and child. For some, staying may have been a calculated effort to keep themselves and their child safe(r) within the constraints of appraised risks and available options. Despite being labeled as failing to protect, not leaving may have been a valiant attempt to better protect.

All recordings were essentially mute on the intersections of socioeconomic status, race, and other subjugated identities, and their impact on the lived experiences of women. It is plausible that poverty and oppression are so naturalized in child protection work that they rarely make it to the page, concealing the social contexts of mothering. Despite known effects on maternal response to CSA, relevant information pertaining to mother–daughter attachment, cultural and religious influences, maternal mental health and trauma, and social supports and coping was absent or glossed over. It is conceivable that struggles were denied or minimized by mothers to avert further scrutiny, perceptions of incompetence, and more invasive interventions. Unvoiced life stories and unspoken problems render needs invisible and goals unattainable. When past and present adversities, individual and structural, are not fully understood, there is predilection to attribute suboptimal protection to the bad-mothering choices of decontextualized women.

## Discussion

This study applied CDA through a feminist lens to challenge dominant interconnected discourses of gender, motherhood, and risk that fuel textually mediated notions of blame and FTP permeating child welfare system responses to the sexual abuse of children. In addition to demonstrating the merits of an underutilized method of interpretive research in violence, my analyses built a credible case for institutional and social change with a strong epistemic and evidentiary foundation for understanding and resisting gendered hierarchies of power in child protection. True to the objectives of critical social science, this discussion considers avenues for remedying the injustices uncovered with the knowledge generated through this critique of policy and practice texts. Although it would seem that massive scale ideological and sociopolitical transformation is a prerequisite for reversing the trends that have come to light, tangible approaches are proposed for working toward surmounting the problems that are anchored in the construction of an alternative discourse with greater coherence and explanatory power.

### Denaturalizing Motherhood Ideology: Embracing Imperfection

This study unearthed enduring cultural constructions of motherhood, the hegemonic effects of which support gender-biased child welfare policies and practices that overtly and covertly fix their gaze on women's mothering while neglecting men's fathering and offending. The sexual abuse of children and the mothering failures of women are so inextricably linked that they have become naturalized. The institutionalized doctrine of FTP emerged as a present-day mutation of historically ubiquitous claims of maternal collusion and complicity in CSA, both implying culpability, constituting blame, and reinforcing patriarchy. Despite societal momentum toward gender equity, women continue to comprise the demographic majority of primary caregivers and victims of violence, and are therefore disproportionately impacted by FTP principles and protocols. Consistent with earlier observations (Davies & Krane, 1996; Lapierre, 2008; Strega et al., 2013; Swift, 1995), the women profiled in this FTP case file analysis inevitably failed to measure up to unrealistic maternal ideals when abstracted from their social context through textual silences and pathologizing representations. Consequently, they were characterized as deficient in their mothering role, worthy of blame, and deserving of punitive repercussions.

Formulating a homogenous model of mothering as natural and universal invites the conclusion that it is superior and unchangeable. Fracturing the social reproduction of motherhood discourse in child welfare begins with exposing and resisting the all-sacrificing, all-knowing, all-powerful maternal archetypes that dichotomize women as good or bad according to where they rank on the yardstick of societal ideals. This does not undermine the feelings of love and desire to nurture and protect most parents have for their child; nor does it negate the existence of harmful mothering. It does, however, challenge essentialist narratives of innately intuitive and omnipotent mothering, and it demands the recontextualization of decontextualized women. Moving away from deficit schemas of mothering that attribute perceived failures in protection to individual inadequacies necessitates consideration of women's lived experiences and material conditions, intersecting sources of oppression, consequences of life-altering decisions, and structural barriers to fulfilling the protection mandate. Rejecting binary thinking that underlies universal applications means accepting the reality of maternal imperfection, embracing diversities among women, making visible the emotional and physical labor of mothering, and replacing discourses that shame and blame with discourses that empower and support. This paradigm shift calls for increased awareness and disruption of unconscious bias among professionals, and policies and practices derived from evidence and experience, not driven by gender normative ideology.

### Redistributing Risk and Responsibility for FTP: Unblaming Mothers

As this study demonstrated, best interests of the child were evaluated against maternal capacity to protect, with perceptions of CSA risk superseding all other considerations. Child welfare formulations of risk were circumscribed to maternal behavior, knowledge, and attitude while perpetrator and environmental risks were textually backgrounded. Risk reduction plans relied on maternal compliance with orders to monitor and control the whereabouts and actions of violent men, thus investing women with a power they did not always possess. Grounded in ideologies of motherhood and neoliberalism, good mothers are rational actors capable of weighing and avoiding risk by way of self-efficacy; bad mothers are ineffectual at circumventing risk due to personal defects or poor choices, rendering themselves deserving of blame and state intervention. Constructing risky mothering as a primary hazard roots the genesis of CSA in maternal acts of omission over perpetrator acts of commission and structural forces. This fundamental blame attribution error emerges from human tendency to assign causal explanations to individual (vs. contextual) variables to gain some semblance of control (Shaver, 1985). The consequences of branding mothers as the embodiment of risk, target of blame, and agent of change were effective offloading of the protection role from criminal justice and child welfare systems (to mothers), reduction of the function of workers to one of surveillance (of mothers), and diversion of liability away from men and gendered power asymmetries (toward mothers).

Findings point to a call for action from policymakers and organizational leaders to adopt a gender-transformative approach to child welfare. This analysis does not abdicate mothers from any or all responsibility for child protection, as such a stance would undermine women's agency and worth, as well as unjustly exonerate those who in fact are abusive. Moreover, all children by nature of their developmental vulnerabilities need and have rights to care and protection. This analysis does argue, however, that risk evaluations of CSA and FTP be firmly grounded in fair principles: (a) intersecting sources of risk should be understood as coalescing at all levels of the ecosystem, not merely beginning and ending with mothers; (b) blame and accountability should be unequivocally situated with those perpetrating abuse against the backdrop of patriarchal culture and institutions, not deflected onto mothers; (c) liabilities for protection should be dispersed among all significant caregivers and mandated systems, not solely relegated to mothers; and (d) expectations for protection should reflect diverse lived experiences and social locations, not idealized scripts of motherhood. The child welfare system, as this study revealed, does not fully subscribe to these tenets, thus necessitating a change in how risk is conceptualized, operationalized, and distributed in ambiguous policies and practice tools. Words matter. A shift in discourse can be set in motion with conscious use of language in active voice that does not minimize, conceal, or obscure gender-based power imbalances and responsibilities. While upholding the goal of reducing probability of harm to the child, technical–rational manifestations of risk-averse discourse demands more tolerance of the uncertainties and nuances intrinsic to protection work and better integration of constructive engagement and critical thinking within an evidence-informed and anti-oppressive framework. Iterating the recommendations of others (Coohey, 2006; Henry et al., 2020; Shadoin & Carnes, 2006) and potential lines for future research, FTP substantiation criteria should be clearly delineated and versed in experiential and empirical knowledge. This entails standards of reasonableness that consider socioemotional, material, and systemic barriers to immediate and enduring protection, both surmountable and insurmountable.

### Disrupting Child-Centrism: Striking a Balance of Best Interests

This study highlighted the polarizing nature of protection policies and practices that pit the rights and needs of children against those of their mothers. Promoting children's best interests is irrefutably important and worthy of codification. The overriding principle, when rigidly applied and ranked above all else, however, invariably works against the interests of women, particularly those who are themselves victims of violence and marginalization (Alaggia et al., 2007). Contesting reductionist discourses of paramountcy in child welfare does not discount the best interests of children; rather, it puts them on equal footing with those of their mothers, thereby diminishing harmfully divisive effects. The tension arising from the dual child welfare mandate was shown in this analysis, where discourses of child protection eclipsed discourses of family support. Toward the quest for healthier balance, sustained emphasis on child safeguarding must be infused with heightened empathy and compassion for maternal adversities. The discovery of CSA is a turbulent process for mothers, often characterized by weakened resources and traumatic stress flooding usual coping faculties. Nonetheless, most mothers act or come to act protectively with support (Bolen, 2002). As this study found, however, there was an unremitting expectation of immediate maternal readiness, willingness, and ability to forgo their own needs and overcome their own crises to fulfill onerous protection duties with little tangible assistance or clinical guidance. The overwhelming stressors epitomizing the everyday lives of mothers were textually overshadowed by incessant orders for behavioral and attitudinal adjustment to the point of self-sacrifice. Despite known relational and contextual influences on maternal response to CSA (Alaggia, 2001; Alaggia & Turton, 2005; Bolen & Lamb, 2002; Wamser-Nanney & Sager, 2018), this analysis uncovered few discernible connections to capacity to protect, with demands for protection persisting under any circumstances and at all costs. Adversarial encounters with CPS appeared to obstruct help-seeking and contribute to resistance, echoing the stigmatization and condemnation voiced by women in other studies (Alaggia, 2002; Plummer & Eastin, 2007a). At the other end of the spectrum of mothers who fail to do enough to protect are those who do too much to protect, eliciting accusations of malicious allegations and parental alienation that create a double bind.

Women's resilience can be strengthened with supportive interventions that target needs both related to and independent of the mothering role, and bolster self-compassion, constructive coping, and social networks (McGillivray et al., 2018; Serin, 2018). An affirming worker–mother relationship, as evident in the negative case analysis, is a potent channel through which children can be better protected. To this end, mindful use of power is essential for respectful and ethical engagement at the core of authentic helping. Relational practice enables safer exploration and negotiation of maternal ambivalence, and repair of emotionally attuned and protective mothering. Capitalizing on strengths and mitigating barriers such as poverty and IPV, two potentially malleable variables imperiled by erasure in the files, would empower mothers immobilized by deprived resources and constrained choices. To the detriment of women and children, IPV risk took a backseat to CSA risk when systems failed to enforce protective orders against violent men. Advancing mother- *and* child-centered discourses in child welfare demands an integrated response that prioritizes the safety and well-being of both without compromising the safety or well-being of either.

### Destabilizing Gendered Practices: Promoting Involved Fatherhood

Sexist occupational culture of child welfare has lagged behind progressive shifts in public discourse surrounding involved fatherhood, as corroborated by this study. This was apparent in what was and, perhaps equally revealing, was not written in the case files. The maternal role in childcare and protection was constructed as compulsory; the involvement of nonoffending fathers was contingent on maternal (in)capacity, and offenders were ostensibly invisible sources of threat under maternal management. This finding confirms well-substantiated CPS failures to actively engage fathers, whether they are risks or resources (Brown et al., 2009; Maxwell et al., 2012a; Strega et al., 2008). A function of the gender role dichotomy, men's avoidance or refusal to engage transpired in the context of ideologically motivated practices that sanctioned their disengagement. Destabilizing maternal primacy requires a paternal-inclusive approach to protection cemented in policy and integrated through all phases of service delivery. As this analysis showed, the adoption of gender-neutral language in child welfare texts, while conducive to inclusivity on the surface, masked mother-centric philosophies and procedures. Top-down attention to lexical choices for the caregiver is fundamental to activating discourses of shared parenting and protecting. Seemingly trivial shifts in semantics provide clout to mandatorily intervene with men, rather than surrender to resistance or succumb to bias. Another practical gateway to change is the restructuring of CPS databases that label and track files under the maternal name by default. This small but feasible reform would detract from the focus on mothers from the outset, at least semiotically and symbolically.

In families with fathers, a positive paternal presence can foster healthy child development and guard against negative effects of adversities (Dubowitz et al., 2001), including abuse (Cyr et al., 2019; Guelzow et al., 2002), although empirical evidence is limited due to the overconcentration of research on mothers. A finding to be embraced with cautious optimism, this analysis uncovered traces of competing discourses promoting involved fatherhood and challenging hegemonic masculinity. Child welfare efforts to engage nonoffending fathers, however, were mostly unsuccessful due to their unwillingness or unpreparedness to provide care, underscoring the need for parenting support. Arming workers with the confidence, attitudes, knowledge, and skills to effectively work with men, particularly violent men who invoke fear or moral discomfort, can serve as an important catalyst for change (Maxwell et al., 2012b). Improved efficacy at the level of the individual may ultimately be futile without a corresponding shift in institutional and cultural consciousness.

## Limitations

The interpretive repertoire and agenda of CDA impart inherent subjectivity in the collection and analysis of data. Although this does not negate methodological soundness or empirical validity, the “gold standard” for research rigor in positivistic methods can never be achieved with this approach. To reduce bias and gain credibility, a number of measures were implemented at each stage of investigation, including goal transparency, data triangulation, thick description, raw text quotes, audit trails, self-reflection, and no claims of absolute truth. In addition to the general criticisms of CDA (Breeze, 2011), limitations specific to text selection and case file review methodology should be considered. All documents were institutionally constructed and socially located. The small sample of files was purposively selected from a single jurisdiction and thus are not representative of the larger population. Accordingly, study findings cannot be generalized beyond the sample. With sole reliance on existing texts, the degree of accuracy of most unilateral recordings was unverifiable, and important verbal and nonverbal elements of speech were inaccessible. Incongruities between the realities of women's lives and their semiotic representations (Smith, 1987) and under-documentation by workers (Strega et al., 2008) are noted shortcomings in file reviews, potentially presenting an incomplete picture of the problem. Study findings are therefore partial and must be interpreted within the boundaries of these limitations.

## Conclusion

Gendered child welfare discourse has proven itself to be remarkably impervious to change. This study effectively problematized and interrupted its stronghold on CSA policy and practice by linking microlevel talk and text to macrolevel ideology and institutional structure that legitimize and reinforce gender-based attributions of blame and FTP in child welfare. Discursive critique gives rise to discursive resistance, negotiation, and transformation. Possibilities for resolve rest on continued critical engagement with prevailing paradigms of gender, motherhood, and risk that function to blame and shame women in isolation of intersecting social locations and material conditions. Moving away from a myopic focus on failed mothering will advance our collective goal of protecting children and dismantling oppressive power relations at the root of the problem.
